# An Informational Test for Random Finite Strings

**DOI:** 10.3390/e20120934

**Published:** 2018-12-06

**Authors:** Vincenzo Bonnici, Vincenzo Manca

**Affiliations:** Department of Computer Science, University of Verona, 37134 Verona, Italy

**Keywords:** algorithmic information theory, incompressibility, typicality, randomness test, pseudo-random generators, k-mer multiplicity, k-entropy, informational indexes

## Abstract

In this paper, by extending some results of informational genomics, we present a new randomness test based on the empirical entropy of strings and some properties of the repeatability and unrepeatability of substrings of certain lengths. We give the theoretical motivations of our method and some experimental results of its application to a wide class of strings: decimal representations of real numbers, roulette outcomes, logistic maps, linear congruential generators, quantum measurements, natural language texts, and genomes. It will be evident that the evaluation of randomness resulting from our tests does not distinguish among the different sources of randomness (natural, or pseudo-casual).

## 1. Introduction

The notion of randomness has been studied since the beginning of probability theory (“Ars Conjectandi sive Stochastice” is the title of the famous treatise of Bernoulli). In time, many connections with scientific concepts were discovered, and random structures have become crucial in many fields, from randomized algorithms to encryption methods. However, very often, the lines of investigation about randomness appeared not to be clearly related and integrated. In the following, we outline a synthesis of the main topics and problems addressed in the scientific analysis of randomness, for a better understanding of the approach presented in the paper.

### 1.1. Mathematical Definitions of Randomness

The attempts to provide mathematical definitions agreeing with the intuitive meaning of this concept have a very long and complex history [1,2]. According to algorithmic information theory [3,4,5,6,7,8,9] a finite string is random if it is incompressible, that is, the length of any program generating it is always longer than the length of the string (decreased by an additive constant independent of the length of the string). This definition opens a wide spectrum of analyses relating randomness to computation and complexity. Algorithmic information theory puts in evidence a strong link relating computation with randomness. This link was also a key point of Martin-Löf’s approach to randomness for infinite strings [10]. In particular, a theoretical notion of the statistical test was developed by him in terms of computability theory. A sequence is defined as random when it passes all recursively-enumerable tests by escaping from fulfilling any computable “peculiar” property of strings. In this sense, a random string has to be typical, or amorphous, or generic. Already, Shannon, in his famous booklet [11] founding information theory, used typicality in the proof of his second theorem, by finding that when the string lengths increase, the number of non-typical sequences vanishes. Just the lack of any peculiarity of typical sequences is responsible for their incompressibility. However, though, incompressibility and typicality represent significant achievements toward the logical analysis of randomness, they suffer in their applicability to real cases. In fact, incompressibility and typicality are not computable properties.

In 1872, Ludwig Boltzmann in the analysis of thermodynamic concepts inaugurated a probabilistic perspective that was the beginning of a new course, changing radically the way of considering physical systems. The following theory of quanta developed by Max Plank [12] can be considered in the same line of thought, and quantum theory as it was defined in the years 1925–1940 [13] and in more recent developments [14] confirms the crucial role of probabilistic and entropic concepts in the analysis of physical reality. According to quantum theory, what is essential in physical observations are the probabilities of obtaining some values when physical variables are measured, that is, physical measurements are random variables, or information sources with peculiar probability distributions, because measurements are random processes. This short mention of quantum mechanics is to stress the importance that randomness plays in physics and in the whole of science and, consequently, the relevance of randomness characterizations for the analysis of physical theories. In this paper, we will show that Shannon entropy, related to Boltzmann’s H function [15], is a key concept in the development of an new approach to the randomness of finite strings.

### 1.2. True Randomness

“True randomness” concerns the more intuitive notion of a random process as it emerges from nature or from games, such as coin tosses, urn extractions, dice, playing cards, and so on. In physics, many phenomena have been discovered, such as Brownian motions of colloidal particles or measurements in quantum systems, where physical parameters are generated without any apparent rule and with a lack of any pattern. The properties of these phenomena are considered random for their total unpredictability or for their chaotic behavior.

For example, let us consider a Bernoulli process [16] generated by extractions from an urn containing the same number of white and black balls that are completely indistinguishable in their physical properties, apart from the color, which has no relevance in the extractions from the urn (blind extractions). If we represent a sequence of extractions from the urn (by inserting again the extracted ball in the urn) by means of a 0/1 sequence where 1 stands for white (success) and 0 for black (failure), then we can say that the Boolean sequence generated in a Bernoulli process is a true random sequence. In fact, the unpredictability of this process is based on the assumption that at any extraction zero of one has the same probability of occurring. For analogous arguments, Brownian motions or quantum systems exploit mathematical models that exhibit analogous kinds of statistical homogeneity. The more appropriate term for both such kinds of randomness is “stochasticity” (from the Greek root with the original meaning of guess). A process where we can only guess events implies the lack of any underlying rule, and only ignorance or uncertainty about this rule, which we do not know because it is secret or because it is too complex to be managed in a reliable way. This is probably the original intuition about randomness.

In stochastic processes, we can define random variables (variables assuming values according to a probability distribution) that follow normal distributions or other distributions related to the normal one. For example, the probability of having *k* times one in a Bernoulli sequence of length *n* is given by a distribution that, for *n* very large, approximates to a normal distribution. Many actual randomness tests are based on an agreement with statistical distributions [17].

### 1.3. Pseudo-Randomness and Deterministic Chaos

A line of research, very important from the applicative point of view, is the theory of pseudo-random generators that is aimed at providing deterministic algorithms generating sequences appearing as true random sequences [17,18], with applications going from bank transaction protocols, to Monte Carlo algorithms, or to cryptography. In [17], a detailed and updated historical account of random number generators was given. Surprisingly, the most part of investigations about the mathematical characterization of randomness (incompressibility and typicality) are unrelated to the practical methods for producing or testing randomness.

In the second part of the 20th Century, within the theory of chaos, another important class of random processes of a deterministic nature was discovered, where, despite determinism, an intrinsic kind of chaos was inherent in their dynamics [19]. Deterministic algorithms were found generating sequences that were hardly distinguishable from true random processes. Today, many classes of deterministic chaotic algorithms are well known and applied in many different contexts. Chaotic dynamics easily transforms into random strings. In fact, numbers are generated according to some number representation; therefore, there are many ways of extracting strings from these dynamics.

### 1.4. Empirical Randomness

In conclusion, we have mathematical randomness (incompressibility, typicality, and more technical specializations of them), true or stochastic randomness, and pseudo-randomness. However, how can we judge, in a safe way, when a given string is random according to the considered definitions? In the line of research inaugurated in [20], there are now a great number of randomness tests [17,18,21,22,23]. Almost all of them are of statistical in nature and implicitly assume the probabilistic nature of true randomness. Therefore, a string is empirically random when it passes a given set of randomness tests. In this case, randomness can be assessed along a spectrum of possibilities related to the number of passed tests and on the specific measures of randomness assigned by each test. Of course, tests show a good level of credibility only if they judge random what the other criteria qualify accordingly.

In the discussion developed so far, we encountered many properties that seem to be related to the intuition of randomness (the etymology of “random” is the Latin root for an asymmetric object for hitting in a very irregular and uncontrolled way). Many other notions and subtle differences could be considered. One of them, individuated by a pioneer in the mathematical analysis of random sequences [17,24], is the periodicity/aperiodicity, or more generally, the repeatability/unrepeatability of proper parts. This aspect is the key point of the analysis that we will develop in the next sections. A sequence of *m* digits is *k*-distributed if any of the $m^{k}$ possible *k*-mers appears with the same frequency in the long run. We say it is *∞*-distributed if it is *k*-distributed for any k>1. Borel [24] called normal to base *m* a real number whose expansion in base *m* is *∞*-distributed. He proved that almost all real numbers (with respect to the uniform measure) are normal to any base. Normality implies that any possible substring occurs infinitely, but in an aperiodic way; therefore, normal numbers are irrational.

Many are the randomness tests that are based on repetitiveness phenomena [17]. However, in this paper, we show that certain precise relationships hold between the length of strings and the lengths of *k*-mers that present some specific properties of repeatability and unrepeatability. In this regard, the values $\log_{m}n$ and $2\log_{m}n$ play a critical role, which can be used for evaluating the randomness of strings of length *n*. Moreover, $2\log_{m}n$ has a natural interpretation in terms of Shannon’s entropy based on the distributions of *k*-mers.

The search for efficient and reliable methods of string randomness evaluation is crucial in computational genomics. Namely, the “divergence” of biological strings from random strings of the same length has resulted in being a powerful method for discovering specific aspects selected by the evolutionary processes of life [25,26,27].

## 2. Informational Indexes of Strings

Given a string α over an alphabet *A* of *m* symbols, then we denote by D(α) the set of all substrings of α and by $D_{k}\left(\alpha \right)$ the set of *k*-mers of α, that is, the strings of D(α) of length *k*. The function $mult_{\alpha }\left(\beta \right)$ gives the number of occurrences of substring β in α. Two important classes of *k*-mers are repeats and hapaxes. The *k*-mer β is a repeat of α if $mult_{\alpha }\left(\beta \right)>1$, whereas β is a hapax of α if $mult_{\alpha }\left(\beta \right)=1$ (the word hapax comes from the Greek root meaning once). In other words, hapaxes are unrepeatable substrings of α.

If β is a repeat of α, then every substring of β is a repeat of α as well. Analogously, if β is a hapax of α, then every string including β as a substring is a hapax of α as well.

In terms of repeats and hapaxes, we can define the following indexes that we call informational indexes because they are associated with a string viewed as the information source in the sense of Shannon’s information theory. These indexes are very useful in understanding the internal structure of strings.

The index:

$$mrl\left(\alpha \right)=\max_{k}\left(\exists w\in D_{k}\left(\alpha \right):mult_{\alpha }\left(w\right)>1\right)$$

(maximum repeat length) is the length of the longest repeats occurring in α, while:

$$mhl\left(\alpha \right)=\min_{k}\left(\exists w\in D_{k}\left(\alpha \right):mult_{\alpha }\left(w\right)=1\right)$$

(minimum hapax length) is the length of the shortest hapaxes occurring in α. Moreover,

$$mcl\left(\alpha \right)=\max_{k}\left(D_{k}\left(\alpha \right)=\Gamma^{k}\right)$$

(maximum complete length) is the maximum length *k* such that all possible *k*-mers occur in α. A straightforward, but inefficient computation of such indexes can be performed in accordance with their mathematical formulation, but more sophisticated manners were implemented in [28] by exploiting suitable indexing data structures.

Indexes LG and 2LG, called logarithmic length and double logarithmic length, are defined by the following equations, where |α| denotes the length of string α (*m* is the number of different symbols occurring in α):

$$LG\left(\alpha \right)=\log_{m}\left(|\alpha |\right)$$

$$2LG\left(\alpha \right)=2\log_{m}\left(|\alpha |\right).$$

When α is given by the context, we simply write: mcl,mhl,mrl,LG,2LG, instead of mcl(α),mhl(α),mrl(α),LG(α),2LG(α), respectively. The following propositions follow immediately from the definitions above.

**Proposition** **1.** *For k>mrl, all k-mer of α are hapaxes.*

**Proposition** **2.** *For k<mhl, all k-mer of α are repeats.*

**Proposition** **3.** *For any string α, mhl≥mcl.*

**Proposition** **4.** *For any string α of length n: $D_{k}\left(\alpha \right)\leq n-k+1$, and if $D_{k}\left(\alpha \right)=n-k+1$, then all the elements of $D_{k}\left(\alpha \right)$ are hapaxes of α.*

**Proposition** **5.** *In any string, the following inequality holds:*

mcl≤⌈LG⌉.

The notation ⌈LG⌉ refers to the ceiling function, namely the smallest integer following the value LG.

By using mult, we can define probability distributions over $D_{k}\left(\alpha \right)$, by setting $p\left(\beta \right)=mult_{\alpha }\left(\beta \right)/\left(|\alpha |-k+1\right)$. The empirical *k*-entropy of the string α is given by Shannon’s entropy with respect to the distribution *p* of *k*-mers occurring in α (we use the logarithm in base *m* for uniformity with the following discussion):

$$E_{k}\left(\alpha \right)=-\sum_{w\in D_{k}\left(\alpha \right)}p\left(w\right)\log_{m}p\left(w\right).$$

It is well known [11] that entropy reaches its maximum for uniform probability distributions. Therefore, when all the *k*-mers of a string α of length *n* occur with the uniform probability 1/(n−k+1), this means that the following proposition holds.

**Proposition** **6.** *If all k-mers of α are hapaxes, then $E_{k}\left(\alpha \right)$ reaches its maximum value in the set of probability distributions over $D_{k}\left(\alpha \right)$.*

## 3. A “Positive” Notion of a Random String

It is not easy to tell when a string is a random string, but it is easy to decide when a given string is not a true random string. A “negative” approach to string randomness could be based on a number of conditions $C_{1},C_{2},\ldots$, each of which implies non-randomness. In this way, when a string does not satisfy any such conditions, we have a good guarantee of its randomness. In a sense, mathematical characterizations of randomness are “negative” definitions based on infinite sets of conditions. Therefore, even if these sets are recursively enumerable, randomness cannot be effectively stated.

Now, we formulate a principle that is a sort of Borel normality principle for finite strings. It expresses a general aspect of any reasonable definition of randomness. In informal terms, this principle says that any substring has the same probability of occurring in a random string, and for the substring under a given length, the knowledge of any prefix of a random string does not give any information about their occurrence in the remaining part of the string. In this sense, a random string is a global structure where no partial knowledge is informative about the whole structure.

**Principle** **1** (Random Log Normality Principle (RLNP)). *In any finite random string α, of length n over m symbols, for any value of k≤n, all possible k-mers have the same a priori probability $1/m^{k}$ of occurring at each position i, for 1≤i≤n−k+1. Moreover, let us define the a posteriori probability that a k-mer occurs at position i as the conditional probability of occurring when the prefix of α[1,i−1] is given. Then, for any k<⌈2LG⌉, at each position i of α, for 1≤i≤n−k+1, the a posteriori probability of any k-mer occurrence has to remain the same as its a priori probability.* □

The reader may wonder about the choice of the ⌈2LG⌉ bound that appears in the RLNP principle stated above. It is motivated by Proposition 7, which is proven by using the first part of the RLNP principle.

In the following, a string is considered to be random if it satisfies the RLNP principle. Let us denote by $RND_{m,n}$ the set of random strings of length *n* over *m* symbols and by RND the union of $RND_{m,n}$ for n,m∈N:

$$RND=\underset{n,m\in N}{⋃}RND_{m,n}.$$

For individual strings, in general, RLNP can hold only admitting some degree of deviance from the theoretical pure case. This means that the randomness of an individual string cannot be assessed as a 0/1 property, but rather, in terms of some measure expressing the closeness to the ideal cases (for example, the percentage of positions where RLNP fails).

According to this perspective, RLNP may not exactly hold in the whole set $RND_{m,n}$. Nevertheless, the subset of $RND_{m,n}$ on which RLNP fails has to approximate to the empty set, or better, to a set of zero measure, as *n* increases. In other words, random strings are “ideal” strings by means of which the degree of similarity to them can be considered as a “degree of membership” of individual strings to RND. This lack of precision is the price to pay for having a “positive” characterization of string randomness.

The following proposition states an inferior length bound for the hapaxes of a random string.

**Proposition** **7.** *For any $\alpha \in RND_{m,n}$, if:*

k≥⌈2LG⌉

*then all k-mers of α are hapaxes of α.*

**Proof.**  Let us consider a length *k* such that:

$$m^{k}\geq n-k+1.$$

According to RLNP, the probability that a *k*-mer occurs in $\alpha \in RND_{m,n}$ is given by the following ratio (the number of available positions of *k*-mers is n−k+1):

(1) $$Prob\left(\alpha \in D_{k}\left(\alpha \right)\right)=\frac{\left(n-k+1\right)}{m^{k}}$$

but, if all *k*-mers are hapaxes in α, then their probability of occurring in α is also given by:

(2) $$Prob\left(\alpha \in D_{k}\left(\alpha \right)\right)=\frac{1}{\left(n-k+1\right)}$$

Then, if we equate the right members of the two equations above (1) and (2), we obtain an equation that has to be satisfied by any length *k*, ensuring that all *k*-mers occurring in α are hapaxes in α:

(3) $${\left(n-k+1\right)}^{2}=m^{k}$$

which implies:

(4) $$2\log_{m}\left(n-k+1\right)=k.$$

In order to evaluate the values of *k*, we solve the equation above by replacing *k* in the left member of Equation (4) by the whole left member of Equation (4):

(5) $$2\log_{m}\left(n-2\log_{m}\left(n-k+1\right)+1\right)=k$$

Now, Equation (5) implies that:

(6) $$2\log_{m}\left(n-2\log_{m}\left(n\right)\right)\leq k\leq 2\log_{m}\left(n\right)$$

However, the difference between the two bounds of *k* is given by:

$$2\log_{m}\left(n\right)-2\log_{m}\left(n-2\log_{m}\left(n\right)\right)=2\log_{m}\frac{n}{n-2\log_{m}\left(n\right)}=2\log_{m}\left(1+\frac{2\log_{m}n}{n-2\log_{m}n}\right)$$

where the right member approximates to zero as *n* increases. In conclusion:

$$k\approx 2\log_{m}\left(n\right).$$

This means that for:

$$k\geq \lceil 2\log_{m}\left(n\right)\rceil =\left\lceil 2LG\right\rceil$$

all *k*-mers of α are hapaxes, that is, ⌈2LG⌉ is a lower bound for all unrepeatable substrings of $\alpha \in RND_{m,n}$. □

The following proposition follows as a direct consequence of the previous proposition and Proposition 1.

**Proposition** **8.** *If α∈RND then:*

mrl+1=⌈2LG⌉.

In conclusion, we have shown that in random strings, 2LG is strictly related to the mrl index.

According to the proposition above, the index 2LG has a clear entropic interpretation: it is the value of *k* such that the empirical entropy $E_{k}$ of a random string reaches its maximum value.

We have seen that in random strings, all substrings longer than ⌈2LG⌉ are unrepeatable, but what about the minimum length of unrepeatable substrings? The following proposition answers this question, by stating an upper bound for the length of repeats in random strings.

**Proposition** **9.** *If α∈RND, then:*

mhl≤⌈LG⌉

**Proof.**  Let us consider k=⌈LG⌉. If some of such *k*-mers is a hapax, the proposition is proven. Otherwise, if no *k*-mer is a hapax of α, then all *k*-mers of α are repeats. However, this fact is against the Random Log Normality Principle (RLNP). Namely, in all the positions *i* of α such that in α[1,i], a *k*-mer β occurs once (these positions necessarily exist), the a posteriori probability that β has of occurring in a position i+1 of α after is 1/(n−k−i), surely greater than $1/m^{k}$ (where n=|α|). Hence, in positions after *i*, the a posteriori probability would be different from the a priori probability. In conclusion, if α∈RND, some hapaxes of length ⌈LG⌉ have to occur in α; thus, necessarily, mhl≤⌈LG⌉. □

**Figure 1:**
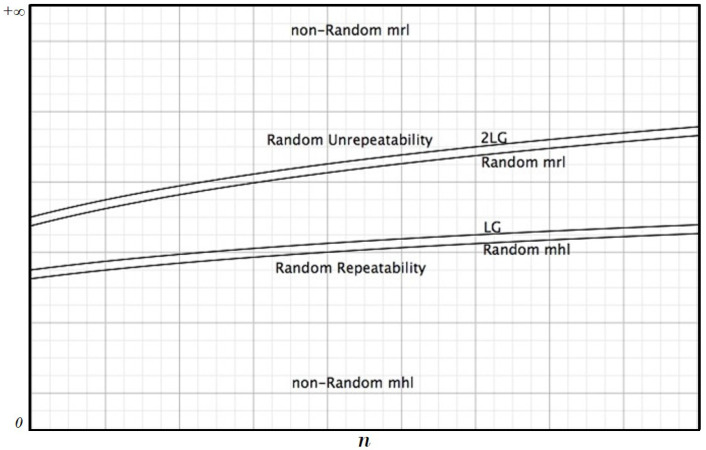
The logarithmic bounds of randomness.

Our analysis shows that in random strings, there are two bounds given by ⌈LG⌉ and ⌈2LG⌉ (Figure 1). Under the first one, we have only repeatable sub-strings, while over the second one, we have only unrepeatable sub-strings. The agreement with these conditions and the degrees of deviance from them give an indication about the randomness degree of a given string.

It is easy to provide examples of strings where these conditions are not satisfied, but what is interesting to remark is that the conditions hold with a very strict approximation for strings that pass the usual randomness statistical tests; moreover, for “long strings”, such as genomes, the bounds hold, but in a very sloppy way because usually mrl+1 is considerably greater than ⌈2LG⌉ and mhl−1 is considerably smaller that ⌈LG⌉.

In our findings reported in Section 5, the best randomness was found for strings of π decimal digits, for quantum measurements, and for strings obtained by pseudo-casual generators [29]. In the next sections, we discuss our experimental data about our randomness parameters.

## 4. Log Bounds Randomness Test

The Log Bounds Test (LBT), based on the analysis developed in the previous sections, essentially checks, for a given string, the logarithmic bounds for its prefixes and in the average (with the standard deviation). We apply LBT to a wide class of strings, in order to verify if it correctly guesses the randomness of strings traditionally judged random and, at the same time, if it does not give such evidence in the cases where it is not appropriate. The results confirm a discrimination capability with a complete accordance with our expectations.

For our analyses, we have taken into account different types of strings that are commonly considered as random strings, available in public archives accessed on June 2018.

A first category of random strings is extracted from a real number such as π and Euler’s constant *e*. In this case, the number is converted into a string over the alphabet of decimal digits, and a given number of digits of the number *e* are extracted. Digits were downloaded from https://www.angio.net/pi/digits/pi1000000.txt and https://apod.nasa.gov/htmltest/gifcity/e.2mil.

Quantum physics-generated data based on the non-determinism of photon arrival times provide up to 150 Mbits/s in the form of a series of bytes, available at http://qrng.physik.hu-berlin.de/.

Another category of random data is given by mathematical functions that provide chaotic dynamics, such as the logistic map with the parameter in [3.8, 4].

Linear congruential generators of the form $x_{n+1}=cx_{n}+b$ generate pseudo-random numbers [17,18] that we converted into strings of suitable alphabets of different sizes by applying discretization mechanisms.

Another category is given by random series related to roulette spins, cards, dice, and Bernoulli urns. In particular, we took into account three million consecutive roulette spins produced by the Spielbank Wiesbaden casino, at the website https://www.roulette30.com/2014/11/free-spins-download.html. As was already recognized by several authors, the randomness of roulette data is not true randomness, and this was also confirmed by our findings.

For comparisons, we used non-random data given by the complete works of Shakespeare available at http://norvig.com/ngrams/shakespeare.txt. These texts were transformed in order to extract from them only letters by discarding other symbols.

We used another comparison text given by the complete genome of the *Sorangium cellulosum* bacterium, downloaded from the RefSeq/NCBI database at https://www.ncbi.nlm.nih.gov/nuccore/NC_010162.1 (with a length of around 13 million nucleotides).

## 5. Analysis of the Experimental Results

When the informational indexes mhl and mrl+1 result in coinciding with LG±1 and 2LG±1, respectively, we consider this coincidence as a positive symptom of randomness, and we will mark this fact by writing ✓ on the right of the compared values (or ✗ in the opposite case). The more these coincidences are found for prefixes of a string, the more the string passes our test (a more precise evaluation could consider not only the number of coincidence, but also how much the values differ, when they do not coincide). As the tables in the next section show, our findings agree, in a very significant way, with the usual randomness/non-randomness expectations. The tables are given for different categories of strings. Informational indexes were computed by a specific platform for long string analysis [28]. In Table 1, from 100,000 up to 30 million decimal expansions of π are considered. The agreement with our theory is almost complete, whence a very high level of randomness is confirmed, with only a very slight deviance.

Other tables are relative to decimal expansions of other real numbers: Table 2 for Euler’s constant, Table 3 for $\sqrt{2}$, and Table 4 for Champernowne’s constant, a real transcendent number obtained by a non-periodic infinite sequence of decimal digits (the concatenated decimal representations of all natural numbers in their increasing order). It is interesting to observe that Euler’s constant and $\sqrt{2}$ have behaviors similar to π, whereas Champernowne’s constant has values indicating an inferior level of randomness.

Table 5 concerns strings coming from the pseudo-casual Java generator (a linear congruential generator). The randomness of these data, with respect to our indexes, is very good.

Table 6 is relative to strings generated via the logistic map. In these cases, randomness is not so clearly apparent, due to a sort of tendency to have patterns of a periodic nature, which agree with the already recognized behaviors due to the limits of computer number representations [30].

Table 7 provides our indexes for quantum data [29], by showing a perfect agreement with a random profile.

Table 8 is relative to three millions of roulette spins, with an almost complete failure of the 2LG-check (even if with a quite limited gap).

Finally, Table 9 and Table 10 are relative to a DNA bacterial genome and to texts of natural language (Shakespeare’s works), respectively. In these cases, as expected, our indexes of randomness reveal low levels of randomness.

## 6. Conclusions

In this paper, we presented an approach to randomness that extends previous results on informational genomics (information theory applied to the analyses of genomes [27]), where the analysis of random genomes was used for defining genome evolutionary complexity [26], and a method for choosing the appropriate k-mer length for discovering sequence similarity in the context of homology relationship between genomes [25]. A general analysis of randomness has emerged that suggests a line of investigation where a theoretical approach is coupled with a practical and experimental viewpoint, as required by the application of randomness to particular situations of interest. Randomness has an intrinsic paradoxical, and at the same time vague, nature. In fact, mathematically-rigorous definitions of randomness are intrinsically uncomputable, and algorithmically-testable properties are not exhaustive.

We introduced the random log normality principle, which resembles Borel’s normality [17], but it is formulated, for finite strings, in terms of a priori and a posteriori probabilities. This principle allows us to define two logarithmic bounds that state precise links between the length of strings and the lengths to which specific phenomena of substring repetitiveness must or cannot hold.

A possible continuation of our investigation could be addressed to extend similar principles and tests for finite structures, such as trees or graphs. In fact, in many applications, it would be very useful to have reliable and simple tests for finite mathematical structures commonly used in data representations.

## Figures and Tables

**Table 1:** Decimal digits of π.

*n*	mhl	⌈LG⌉	Check	mrl+1	⌈2LG⌉	Check
100,000	4	5	✓	10	10	✓
1,000,000	5	6	✓	13	12	✓
2,000,000	6	7	✓	13	14	✓
5,000,000	6	7	✓	13	14	✓
10,000,000	6	7	✓	15	14	✓
20,000,000	7	8	✓	15	16	✓
50,000,000	7	8	✓	16	16	✓

**Table 2:** Decimal digits of Euler’s constant *e*.

*n*	mhl	⌈LG⌉	Check	mrl+1	⌈2LG⌉	Check
100,000	4	5	✓	10	10	✓
200,000	5	6	✓	12	12	✓
500,000	5	6	✓	12	12	✓
1,000,000	5	6	✓	13	12	✓
1,200,000	5	7	✗	13	14	✓
1,500,000	6	7	✓	13	14	✓
2,000,000	6	7	✓	13	14	✓

**Table 3:** Decimal digits of $\sqrt{2}$.

*n*	mhl	⌈LG⌉	Check	mrl+1	⌈2LG⌉	Check
10,000	4	4	✓	8	8	✓
20,000	4	5	✓	8	10	✗
50,000	4	5	✓	10	10	✓
100,000	4	5	✓	11	10	✓
200,000	5	6	✓	11	12	✓
500,000	5	6	✓	11	12	✓
1,000,000	5	6	✓	12	12	✓

**Table 4:** Decimal digits of Champernowne’s constant.

*n*	mhl	⌈LG⌉	Check	mrl+1	⌈2LG⌉	Check
10	1	2	✓	2	3	✓
100	2	2	✓	3	4	✓
1000	3	3	✓	6	6	✓
10,000	4	4	✓	9	8	✓
100,000	5	5	✓	12	10	✗
1,000,000	5	6	✓	15	12	✓
10,000,000	6	7	✓	18	14	✗

**Table 5:** Pseudo-random decimal numbers generated by the Java linear congruential generator.

*n*	mhl	⌈LG⌉	Check	mrl+1	⌈2LG⌉	Check
100	2	2	✓	4	4	✓
1000	3	3	✓	6	6	✓
10,000	4	4	✓	8	8	✓
100,000	4	5	✓	10	10	✓
1,000,000	5	6	✓	12	12	✓
10,000,000	6	7	✓	14	14	✓

**Table 6:** Strings generated by logistic maps with seed 0.1 and parameter r=4. Generated numbers are normalized in the interval (0,1) and thus discretized into 10 and 1000 digits.

*n*	mhl	⌈LG⌉	Check	mrl+1	⌈2LG⌉	Check
Alphabet size 10						
10	1	2	✓	2	4	✗
50	1	2	✓	7	4	✗
100	1	2	✓	10	4	✗
200	2	3	✓	11	6	✗
500	2	3	✓	14	6	✗
1000	2	3	✓	17	6	✗
10,000	2	4	✗	21	8	✗
100,000	2	5	✗	32	10	✗
1,000,000	2	6	✗	35	12	✗
5,000,000	2	7	✗	39	14	✗
10,000,000	2	7	✗	42	14	✗
52,000,000	2	8	✗	61	16	✗
Alphabet size 1000						
10	1	2	✓	2	4	✗
50	1	2	✓	3	4	✓
100	1	2	✓	3	4	✓
200	1	2	✓	5	4	✓
500	1	2	✓	8	4	✗
1000	1	2	✓	8	4	✗
10,000	1	2	✓	18	4	✗
100,000	2	2	✓	25	4	✗
1,000,000	2	2	✓	28	4	✗
5,000,000	2	3	✓	34	6	✗
10,000,000	2	3	✓	38	6	✗
52,000,000	2	3	✓	52	6	✗

**Table 7:** Raw quantum data (alphabet size 256).

*n*	mhl	⌈LG⌉	Check	mrl+1	⌈2LG⌉	Check
100,000	2	3	✓	5	6	✓
500,000	2	3	✓	6	6	✓
1,000,000	3	3	✓	6	6	✓
5,000,000	3	3	✓	6	6	✓
10,000,000	3	3	✓	7	6	✓
50,000,000	3	4	✓	7	8	✓

**Table 8:** Roulette spins (alphabet size 37).

*n*	mhl	⌈LG⌉	Check	mrl+1	⌈2LG⌉	Check
100,000	3	4	✓	7	8	✓
200,000	3	4	✓	31	8	✗
500,000	3	4	✓	31	8	✗
1,000,000	4	4	✓	315	8	✗
1,500,000	4	4	✓	315	8	✗
2,000,000	4	5	✓	315	10	✗
2,500,000	4	5	✓	315	10	✗
3,000,000	4	5	✓	315	10	✗

**Table 9:** *Sorangium cellulosum*’s genome (alphabet size 4).

*n*	mhl	⌈LG⌉	Check	mrl+1	⌈2LG⌉	Check
100	2	4	✗	8	7	✓
1000	3	5	✗	14	10	✗
10,000	4	7	✗	17	14	✗
100,000	5	9	✗	116	17	✗
1,000,000	6	10	✗	381	20	✗
10,000,000	7	12	✗	2,721	24	✗
13,033,770	7	12	✗	2,721	24	✗

**Table 10:** Shakespeare’s collection (alphabet size 26).

*n*	mhl	⌈LG⌉	Check	mrl+1	⌈2LG⌉	Check
10,000	1	3	✗	25	6	✗
100,000	2	4	✗	42	8	✗
200,000	2	4	✗	117	8	✗
500,000	2	5	✗	287	10	✗
1,000,000	2	5	✗	287	10	✗
1,500,000	2	5	✗	287	10	✗
2,000,000	2	5	✗	287	10	✗
2,500,000	2	5	✗	287	10	✗
3,000,000	2	5	✗	286	10	✗
3,301,740	2	5	✗	286	10	✗
